# Analysis of Monocytic and Granulocytic Myeloid-Derived Suppressor Cells Subsets in Patients with Hepatitis C Virus Infection and Their Clinical Significance

**DOI:** 10.1155/2015/385378

**Published:** 2015-03-01

**Authors:** Gang Ning, Lanhui She, Lirong Lu, Ying Liu, Yingfu Zeng, Ying Yan, Chaoshuang Lin

**Affiliations:** ^1^Department of Infectious Diseases, The Third Affiliated Hospital of Sun Yat-Sen University, Guangzhou 510630, China; ^2^Intranet of Guangzhou Woman and Children's Medical Center, Guangzhou 510630, China; ^3^Department of Infectious Diseases, First Affiliated Hospital of Kunming Medical University, Yunnan, Kunming 650032, China

## Abstract

Myeloid-derived suppressor cells (MDSCs) have been shown to inhibit T-cell responses in many diseases, but, in hepatitis C virus (HCV) infected patients, MDSCs are still poorly studied. In this assay, we investigated the phenotype and frequency of two new populations of MDSCs denoted as monocytic and granulocytic MDSCs (M-MDSCs and G-MDSCs) in HCV infected patients and analyzed their clinical significance in these patients respectively. We found that the frequency of CD14^+^HLA-DR^−/low^ cells (M-MDSCs) from HCV infected patients (mean ± SE, 3.134% ± 0.340%) was significantly increased when compared to healthy controls (mean ± SE, 1.764% ± 0.461%) (*Z* = −2.438,* P* = 0.015), while there was no statistical difference between the frequency of HLA-DR^−/low^CD33^+^CD11b^+^CD15^+^ (G-MDSCs) of HCV infected patients and healthy donors (0.201% ± 0.038% versus 0.096% ± 0.026%,* P* > 0.05), which suggested that HCV infection could cause the proliferation of M-MDSCs instead of G-MDSCs. Besides, we found that the frequency of M-MDSCs in HCV infected patients had certain relevance with age (*r* = 0.358,* P* = 0.003); patients older than 40 years old group (mean ± SE, 3.673% ± 0.456%) had a significantly higher frequency of M-MDSCs than that of age less than 40 years old group (mean ± SE, 2.363% ± 0.482%) (*Z* = −2.685,* P* = 0.007). The frequency of M-MDSCs, however, had no correlation with HCV RNA loads, aspartate aminotransferase (AST), alanine aminotransferase (ALT), and the level of liver inflammation degree.

## 1. Introduction

Several studies have shown that persistent HCV infection, which leads to the development of chronic hepatitis C (CHC) or hepatocellular carcinoma (HCC), was associated with impaired T cell responses. It is widely accepted that host immune injury, particularly the impaired T cell responses, plays an important role in HCV persistent infection [1–3]. It has been reported that the weakened virus-specific CD4+ and CD8+ T cells responses are associated with disabled antigen presentation by dendritic cells (DCs) [4], abnormal increased regulatory T cells (Tregs) [5, 6], high expressed programmed death 1 (PD-1) [7], and T cell immunoglobulin and mucin domain 3 (Tim-3) [8]. However, the precise inhibitory mechanisms responsible for primary T cell failure or T cell exhaustion are still unclear. MDSCs are a heterogeneous cell population which plays a crucial role in negative regulate of immune responses [9]. In vitro experiments, MDSCs have also been shown to inhibit T-cell activation and proliferation and promote their apoptosis [10]. Some studies have also shown that MDSCs can suppress T cell responses via overexpressed arginase-I or reactive oxygen species (ROS) production and thus facilitate HCV persistent infection [11, 12]. Human MDSCs express the common myeloid marker CD33 but lack of the expression of mature myeloid marker HLA-DR. Besides, it has been suggested that MDSCs are usually divided into monocytic and granulocytic subsets based on the expression of CD14 or CD15 [9]. In this study, we analyzed the distribution differences between M-MDSCs and G-MDSCs in peripheral blood mononuclear cells (PBMC) of HCV infected patients and aim to explore the clinical significance of each subset in these patients

## 2. Subjects and Methods

### 2.1. Study Population

A total of 68 treatment-naive patients with HCV were enrolled from the Third Affiliated Hospital of Sun Yat-Sen University (Guangzhou, China) from April 2012 to July 2010. The population recruited in this study was composed of three groups of subjects, including 56 CHC patients and 12 patients of hepatitis C related liver cirrhosis; 15 healthy controls were randomly selected from the medical center of the Third Affiliated Hospital of Sun Yat-Sen University. All the detailed characteristics of study subjects are presented in Table 1. The exclusion criteria for our study included patients who were (1) taking antiviral therapy or immunosuppressive agents in recent six months; (2) coinfected with HAV, HBV, HDV, HEV or human immunodeficiency virus (HIV), autoimmune diseases (such as hyperthyroidism, diabetes, or autoimmune hepatitis), and any other known cause of liver disease; (3) pregnant or nursing women; (4) with psychiatric disorders; (5) with malignancy.

### 2.2. Ethics Statement

The study protocol was approved by the Ethics Review Board of the Third Affiliated Hospital of Sun Yat-Sen University. Written informed consent was obtained from the patients or their families prior to enrollment.

### 2.3. Peripheral Blood Mononuclear Cells (PBMC) Isolation and Storage

Peripheral blood was drawn (10 mL) into EDTA anticoagulation tubes (Invitrogen, BD) from healthy controls and patients with HCV. Peripheral blood mononuclear cells (PBMCs) were isolated by Ficoll (Amersham Biosciences) density gradient centrifugation within 4 hours. Cells were washed in RPMI 1640 media (Invitrogen, Grand Island, NY) twice and then resuspended in freeze medium (90% FBS (Life Technologies) and 10% DMSO (Sigma-Aldrich, St. Louis, MO)). Finally, PBMCs were transferred into cryovials (1 mL vial-1), cryopreserved at −80°C, and 72 hours later transferred to the liquid nitrogen.

### 2.4. Cryopreservation and Thawing

For analysis, cryovials were removed from the liquid nitrogen, and then were put into the 37°C water bath thawing quickly within 1 min. Then, the cells were resuspended in 10 mL of complete medium (90% RPMI 1640 media, 10% FBS). After being washed twice, the cells were counted by a light microscope after trypan blue dye staining, and then we resuspended the cells and adjusted the concentration to 1 × 10^6^ cells/mL by complete medium.

### 2.5. Flow Cytometry

To determine the frequency and phenotype of CD14^+^HLA-DR^−/low^ (M-MDSCs) and HLA-DR^−/low^CD33^+^CD11b^+^CD15^+^ (G-MDSCs) cells in PBMCs, the following labeled multicolor fluorescence anti-human monoclonal antibodies (mAbs) were used for surface staining: anti-HLA-DR-FITC, anti-CD33-APC, anti-CD11b-PE, anti-CD15-PERCP-Cy5.5, and anti-CD14-PERCP-Cy5.5 (BD Pharmingen, USA). The cells of each sample were incubated with these labeled multicolor fluorescence anti-human monoclonal antibodies at 4°C for 30 minutes. After surface staining, the cells were washed with 2 mL flow staining buffer (PBS plus 1% FBS) at 4°C and centrifuged at 400 g for 5 minutes. Cell pellets were diluted in 300 *μ*L PBS after the supernatant was removed. Flow cytometry was done on a FACSCalibur. All FACS-data was analyzed by FlowJo software (TreeStar, Inc., Ashland, OR). Isotype-matched control antibodies were used as negative controls.

### 2.6. Measurement of Viral Load, Liver Function

Plasma HCV RNA was quantified by reverse transcriptase polymerase chain reaction assay (DAAN Gene, Sun Yat-Sen University, China). AST and ALT were tested in the laboratory center of our hospital with Hitachi7170 automatic biochemistry analyzer.

### 2.7. Statistical Analysis

All data were analyzed by SPSS Statistics 17.0 and all figures were made by Prizm 5.0 statistical analysis software (GraphPad Software). Quantitative data normally distributed are expressed as mean ± standard, followed by the *t* test of means between groups. For nonnormally distributed data, variables are expressed as medians, interquartile range, and the nonparametric Mann-Whitney *U* tests were used to compare differences between groups. The correlation between the frequency of MDSCs and patients clinical parameters was analyzed by Spearman's rank tests, and *P* value less than 0.05 was considered to be statistically significant.

## 3. Results

### 3.1. MDSCs Subsets Frequency in the Peripheral Blood of HCV Patients

**Figure 1:**
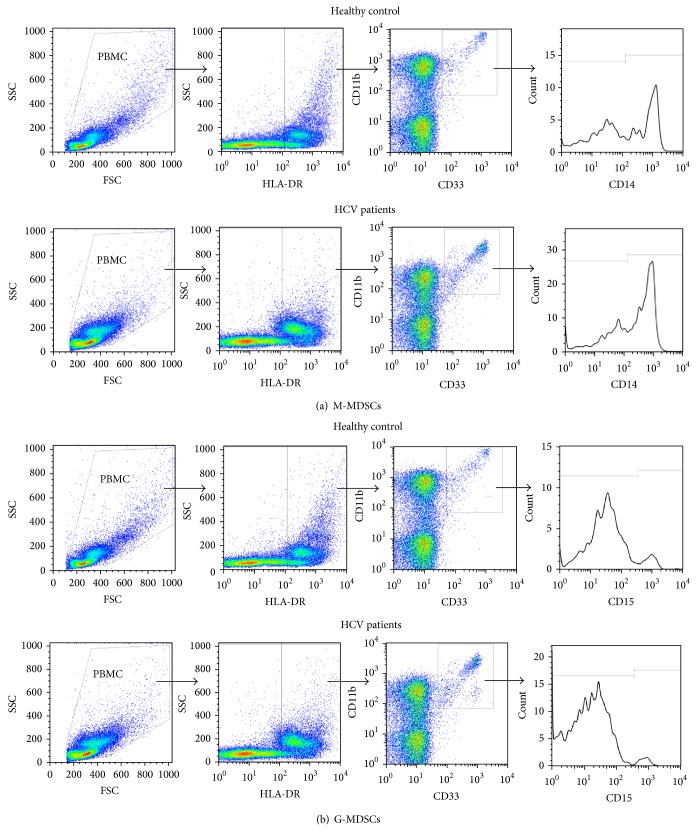
Phenotypic analysis of MDSCs subsets in patients with HCV infection. PBMCs were obtained from patients (*n* = 68) and healthy control (*n* = 15). Fluorochrome-labeled antibodies targeting CD33, HLA-DR, CD15, CD14, and CD11b or the appropriate isotype controls were used to stained MDSCs. Then, MDSCs levels were evaluated by flow cytometry. (a) Flow cytometry analysis was performed with gates set on CD14^+^HLA-DR^−/low^ cells populations (M-MDSCs). (b) Flow cytometry analysis was performed with gates set on HLA-DR^−/low^CD33^+^CD11b^+^CD15^+^ cells populations (G-MDSCs).

**Figure 2:**
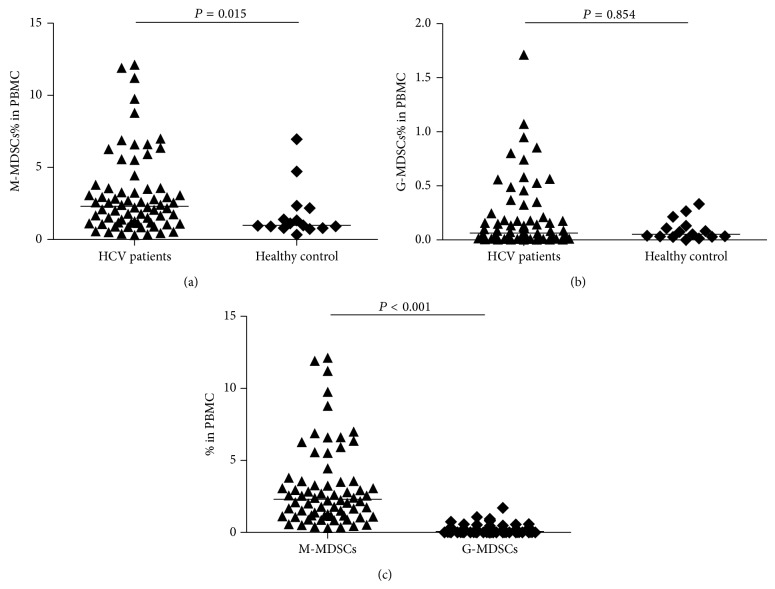
Frequency of two MDSCs subpopulation in HCV patients and healthy control. (a) M-MDSCs from 68 HCV patients and 15 healthy donors. (b) G-MDSCs from 68 HCV patients and 15 healthy donors. (c) Frequency of M-MDSCs and G-MDSCs population in PMBC of HCV patients.

To analyze circulating MDSCs subsets in patients of HCV infection, we first analyzed the expression differences of M-MDSCs and G-MDSCs between HCV infected patients and healthy volunteers. Representative flow cytometry plots of these two distinct cell populations were presented in Figures 1(a) and 1(b). Results clearly reflect that the percentage of M-MDSCs in patients of HCV infection were markedly expanded when compared to healthy individuals (2.305% versus 0.978%, *P* = 0.015) (Figure 2(a)), while there was no statistical difference between the frequency of G-MDSCs in HCV infected patients and healthy volunteers (0.064% versus 0.052, *P* > 0.05) (Figure 2(b)). What is more is that the expression of M-MDSCs was significantly higher than G-MDSCs in HCV infected patients (2.305% versus 0.064%, *P* < 0.001) (Figure 2(c)).

### 3.2. The Frequency of M-MDSCs in CHC and Hepatitis C Related Liver Cirrhosis Patients

**Figure 3:**
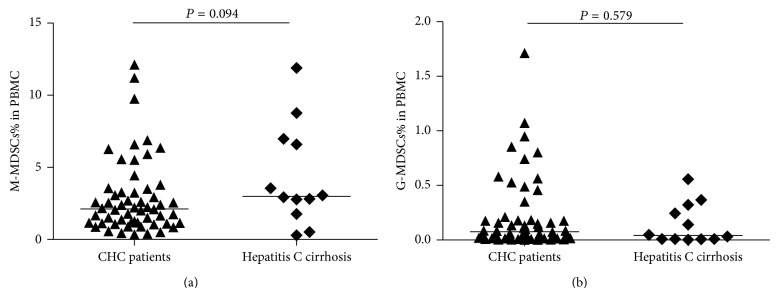
Frequency of two MDSCs subpopulation in CHC patients compared to hepatitis C cirrhosis patients. (a) M-MDSCs from 56 CHC patients and 12 hepatitis C cirrhosis patients. (b) G-MDSCs of 56 CHC patients and 12 hepatitis C cirrhosis patients.

HCV patients were divided into CHC groups and hepatitis C related liver cirrhosis groups based on their different disease progression. The frequency of M-MDSCs in liver cirrhosis patients was higher than CHC patients, but there was no statistical significance (2.995% versus 2.120%, *P* = 0.094) (Figure 3(a)). However, the frequency of G-MDSCs in HCV infected patients was higher when compared to liver cirrhosis patients, but there was also no statistical significance (0.076% versus 0.041%, *P* = 0.579) (Figure 3(b)).

### 3.3. The Correlation of M-MDSCs Expression with Clinical Parameters

**Figure 4:**
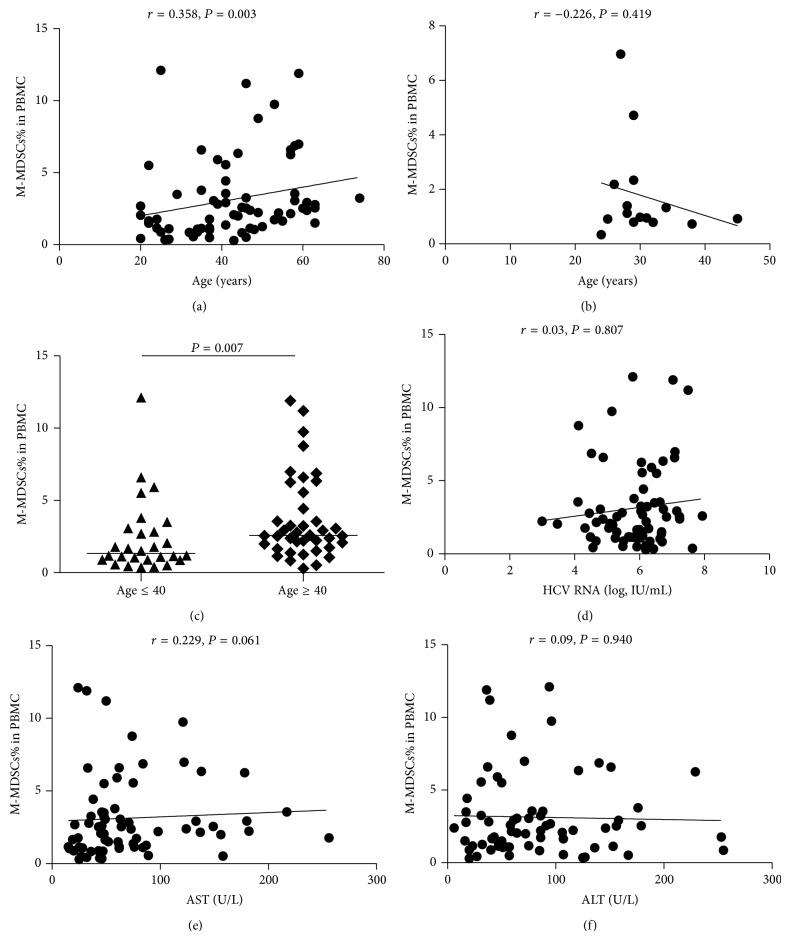


We also determined whether the increased frequency of MDSCs in HCV infected patients was associated with their clinical parameters (Table 1). Interestingly, we found that the frequency of M-MDSCs was positively correlated with the age (*r* = 0.358, *P* = 0.003) (Figure 4(a)) compared with healthy volunteers (*r* = −0.226, *P* = 0.419) (Figure 4(b)). Then we divided them into age ≤ 40 yr group and age ≥ 40 yr group and found the frequency of M-MDSCs of the former group was significantly higher than that of the latter group (2.580% versus 1.330%, *P* = 0.007) (Figure 4(c)). In addition, the percentage of M-MDSCs did not show a correlation with the HCV RNA load (*r* = −0.038, *P* = 0.756) (Figure 4(d)). Also, No correlation was observed between M-MDSCs and AST (*r* = 0.222, *P* = 0.069) (Figure 4(e)) or ALT (*r* = 0.133, *P* = 0.279) (Figure 4(f)).

## 4. Discussion

MDSCs were involved in the immune tolerance of various diseases, such as cancers, autoimmune diseases, and acute and chronic infection diseases [13–15], and played an important part in suppressing the host's natural and adaptive immune responses, especially the T cell responses. Recent researches have reported that the percentage of MDSCs increased in chronic HCV infected patients and might be related to persistent HCV infection [11, 12, 16]. Hitherto, most of the studies about MDSCs including in HCV infection were based on mixed cell levels and only a few of them focused on the function of each subtype of MDSCs. In this study, we distinguished two distinct phenotype cells (M-MDSCs and G-MDSCs), and only the M-MDSCs were found to accumulate during HCV infection.

In tumors, two main subsets of MDSCs were identified. One was referred to as M-MDSCs with the expression of CD14, which have a common phenotype and morphology but distinct function compared to monocytes. The other was G-MDSCs with the expression of CD15, which have common phenotype and significant suppressive activity compared to granulocytes [9, 17]. Interestingly, both M-MDSCs and G-MDSCs have potent T cell suppressive activity via distinct effector molecules and signaling pathways. Some studies indicated that patients with renal cell carcinoma (RCC), colon carcinoma, and lung cancer have increased numbers of G-MDSCs [18–20], while M-MDSCs was the main subpopulation in patients of melanoma, squamous cell carcinoma of the head and neck, pancreatic cancer, hepatocellular carcinoma, and multiple myeloma patients [19, 21–24]. One study revealed that the suppressive activity of M-MDSCs was associated with increased activity of arginase, increased differentiation, and proliferation of Tregs, but without TGF-*β* secretion [17]. However, another contrary result which was found in the research of RCC patients showed that the suppressive activity of M-MDSCs was mediated by TGF-*β* and had nothing to do with the activity of arginase [25].

In this study, we observed a significant elevation of M-MDSCs in peripheral blood of HCV infected patients compared to healthy controls. What is more is that the frequency of M-MDSCs was also higher than G-MDSCs in HCV infected patients, while the expression of G-MDSCs in HCV patients was as low as healthy controls. These results suggested that HCV infection could cause the proliferation of M-MDSCs. As we all know the density gradient centrifugation has been a traditional method to separate PBMCs, such as lymphocytes and monocytes, from polymorphonuclear cells. Most granulocytes, mature CD15 positive cells, deposit to the cell pellet after Ficoll centrifugation because of their high density. Nevertheless, it is possible that some granulocytes with slightly lower density persisted and accounted for the HLA-DR^−/low^CD33^+^CD11b^+^CD15^+^ MDSCs [26–28]. Besides, a study found that CD15 positive subsets of MDSCs were significantly decreased after the cryopreservation or thawing procedure compared to the CD14 positive subsets [29], which would lead to the relatively low proportion of G-MDSCs compared to M-MDSCs in this study. To get more information about those two different subsets, we will do further researches to explore the frequency and clinical significance of M-MDSCs and G-MDSCs by using the whole blood after lysing the red blood cells.

HCV is prone to cause persistent infection and result in HCV related cirrhosis. In addition to the virus mutation, the impaired immune system plays a main role in liver damage. The frequency of M-MDSCs or G-MDSCs in CHC patients was nearly equivalent to CHC patients with liver cirrhosis in this study, which might indicate that these two MDSCs subsets make little sense to the disease progression from chronic CHC to cirrhosis. What is more is that we found that the frequency of M-MDSCs was associated with the age of patients compared to healthy volunteers. Then we divided the HCV patients into age > 40 yr group and age ≤ 40 yr group and found that M-MDSCs in the former group was significantly higher than that of the latter group. It may be related to the duration of HCV infection. As it has found that the younger age of the patients, the shorter duration they may be infected. In other studies, It has been reported that the frequency of M-MDSCs is correlated with the clinical biochemical parameters of HCV patients, including HCV viral load and the level of ALT and AST which reflect liver injury [12, 30]. However, we found no correlation between the frequency of M-MDSCs and HCV RNA, AST, or ALT. We speculated that the main cause would be the limited number of patients; moreover, the transaminase of most patients was close to normal level. Therefore, more patients should be recruited in further studies. As there was no obvious correlation between the level of M-MDSCs with HCV RNA load and it appeared that the increased number of M-MDSCs might be related to the immune response caused by the active viral replication rather than the virus itself, it still needs further studies on the correlation among the accumulation of MDSCs in peripheral blood, cytokines which reflect inflammation and host immune status.

With the complexity and heterogeneity of MDSCs, it is difficult to accurately analyze the phenotype of MDSCs subpopulations in human and their clinical significance. Here we have demonstrated a significant accumulation of M-MDSCs rather than G-MDSCs in peripheral blood of HCV infected patients and we also explored the clinical significance of M-MDSCs and G-MDSCs in HCV infected patients. This observation may help to shed light on the possible role of M-MDSCs in HCV infected patients and lay a foundation for the immune targeted therapy to hepatitis C.

## Figures and Tables

**Table 1:** Basic characteristics of subjects.

Index	HCV patients (*n* = 68)	CHC patients (*n* = 56)	Hepatitis C cirrhosis (*n* = 12)	Healthy control (*n* = 15)
Sex (male/female)	45/23	34/22	11/1	7/8
Age (years, *X* ± *S*)	42.66 ± 13.17	41.20 ± 13.40	49.33 ± 9.89	30.30 ± 5.40
HCV RNA (Iog IU/mL, mean ± SE)	5.847 ± 0.122	6.017 ± 0.132	5.053 ± 0.313	NA
HCV genotype				
1b		20 (29.4%)		NA
6a		37 (54.4%)		NA
others		11 (16.2%)		NA
AST (U/L, mean ± SE)	72.71 ± 6.29	65.18 ± 5.57	107.83 ± 22.44	18.60 ± 6.68
ALT (U/L, mean ± SE)	82.96 ± 6.89	84.64 ± 7.29	75.08 ± 19.75	23.2 ± 5.78

NA: not applicable.

## Abbreviations

MDSCs: Myeloid-derived suppressor cells
HCV: Hepatitis C virus
G-MDSCs: Granulocytic myeloid-derived suppressor cells
M-MDSCs: Monocytic myeloid-derived suppressor cells
CHC: Chronic hepatitis C
HCC: Hepatocellular carcinoma
DCs: Dendritic cells
Tregs: Regulatory T cells
PD-1: Programmed death 1
Tim-3: T cell immunoglobulin and mucin domain 3
ROS: Reactive oxygen species
PBMC: Peripheral blood mononuclear cells
HIV: Human immunodeficiency virus
RCC: Renal cell carcinoma.

## References

[1] Bowen D. G., Walker C. M. (2005). Adaptive immune responses in acute and chronic hepatitis C virus infection. *Nature*.

[2] Rehermann B. (2009). Hepatitis C virus versus innate and adaptive immune responses: a tale of coevolution and coexistence. *The Journal of Clinical Investigation*.

[3] Schulze zur Wiesch J., Ciuffreda D., Lewis-Ximenez L. (2012). Broadly directed virus-specific CD4+ T cell responses are primed during acute hepatitis C infection, but rapidly disappear from human blood with viral persistence. *Journal of Experimental Medicine*.

[4] Ryan E. J., O'Farrelly C. (2011). The affect of chronic hepatitis C infection on dendritic cell function: a summary of the experimental evidence. *Journal of Viral Hepatitis*.

[5] Rushbrook S. M., Ward S. M., Unitt E. (2005). Regulatory T cells suppress in vitro proliferation of virus-specific CD8^+^ T cells during persistent hepatitis C virus infection. *Journal of Virology*.

[6] Cabrera R., Tu Z., Xu Y. (2004). An immunomodulatory role for CD4^+^CD25^+^ regulatory T lymphocytes in hepatitis C virus infection. *Hepatology*.

[7] Golden-Mason L., Palmer B., Klarquist J., Mengshol J. A., Castelblanco N., Rosen H. R. (2007). Upregulation of PD-1 expression on circulating and intrahepatic hepatitis C virus-specific CD8^+^T cells associated with reversible immune dysfunction. *Journal of Virology*.

[8] Golden-Mason L., Palmer B. E., Kassam N. (2009). Negative immune regulator Tim-3 is overexpressed on T cells in hepatitis C virus infection and its blockade rescues dysfunctional CD4^+^ and CD8^+^ T cells. *Journal of Virology*.

[9] Gabrilovich D. I., Nagaraj S. (2009). Myeloid-derived suppressor cells as regulators of the immune system. *Nature Reviews Immunology*.

[10] Nagaraj S., Schrum A. G., Cho H. I., Celis E., Gabrilovich D. I. (2010). Mechanism of T cell tolerance induced by myeloid-derived suppressor cells. *Journal of Immunology*.

[11] Tacke R. S., Lee H.-C., Goh C. (2012). Myeloid suppressor cells induced by hepatitis C virus suppress T-cell responses through the production of reactive oxygen species. *Hepatology*.

[12] Cai W., Qin A., Guo P. (2013). Clinical significance and functional studies of Myeloid-derived suppressor cells in chronic Hepatitis C patients. *Journal of Clinical Immunology*.

[13] Wu W.-C., Sun H.-W., Chen H.-T. (2014). Circulating hematopoietic stem and progenitor cells are myeloid-biased in cancer patients. *Proceedings of the National Academy of Sciences of the United States of America*.

[14] Wu P., Wu D., Ni C. (2014). *γδ*T17 cells promote the accumulation and expansion of myeloid-derived suppressor cells in human colorectal cancer. *Immunity*.

[15] Lu L. R., Liu J., Xu Z. (2014). Expression and clinical significance of myeloid derived suppressor cells in chronic hepatitis B patients. *Asian Pacific Journal of Cancer Prevention*.

[16] Liu Y., She L.-H., Wang X.-Y. (2014). Expansion of myeloid-derived suppressor cells from peripheral blood decreases after 4-week antiviral treatment in patients with chronic hepatitis C. *International Journal of Clinical and Experimental Medicine*.

[17] Gabrilovich D. I., Ostrand-Rosenberg S., Bronte V. (2012). Coordinated regulation of myeloid cells by tumours. *Nature Reviews Immunology*.

[18] Rodriguez P. C., Ernstoff M. S., Hernandez C. (2009). Arginase I-producing myeloid-derived suppressor cells in renal cell carcinoma are a subpopulation of activated granulocytes. *Cancer Research*.

[19] Mandruzzato S., Solito S., Falisi E. (2009). IL4R*α*
^+^ myeloid-derived suppressor cell expansion in cancer patients. *Journal of Immunology*.

[20] Liu C.-Y., Wang Y.-M., Wang C.-L. (2010). Population alterations of L-arginase- and inducible nitric oxide synthase-expressed CD11b^+^/CD14^−^/CD15^+^/CD33^+^ myeloid-derived suppressor cells and CD8^+^T lymphocytes in patients with advanced-stage non-small cell lung cancer. *Journal of Cancer Research and Clinical Oncology*.

[21] Serafini P., Meckel K., Kelso M. (2006). Phosphodiesterase-5 inhibition augments endogenous antitumor immunity by reducing myeloid-derived suppressor cell function. *Journal of Experimental Medicine*.

[22] Vuk-Pavlović S., Bulur P. A., Lin Y. (2010). Immunosuppressive CD14^+^HLA-DR^low/-^ monocytes in prostate cancer. *Prostate*.

[23] Hoechst B., Ormandy L. A., Ballmaier M. (2008). A new population of myeloid-derived suppressor cells in hepatocellular carcinoma patients induces CD4^+^CD25^+^Foxp3^+^ T cells. *Gastroenterology*.

[24] Brimnes M. K., Vangsted A. J., Knudsen L. M. (2010). Increased level of both CD4+FOXP3+ Regulatory T Cells and CD14+HLADR−/low myeloid-derived suppressor cells and decreased level of dendritic cells in patients with multiple myeloma. *Scandinavian Journal of Immunology*.

[25] Filipazzi P., Valenti R., Huber V. (2007). Identification of a new subset of myeloid suppressor cells in peripheral blood of melanoma patients with modulation by a granulocyte-macrophage colony-stimulation factor-based antitumor vaccine. *Journal of Clinical Oncology*.

[26] Rodrigues J. C., Gonzalez G. C., Zhang L. (2010). Normal human monocytes exposed to glioma cells acquire myeloid-derived suppressor cell-like properties. *Neuro-Oncology*.

[27] Pillay J., Tak T., Kamp V. M., Koenderman L. (2013). Immune suppression by neutrophils and granulocytic myeloid-derived suppressor cells: similarities and differences. *Cellular and Molecular Life Sciences*.

[28] Schmielau J., Finn O. J. (2001). Activated granulocytes and granulocyte-derived hydrogen peroxide are the underlying mechanism of suppression of T-cell function in advanced cancer patients. *Cancer Research*.

[29] Kotsakis A., Harasymczuk M., Schilling B., Georgoulias V., Argiris A., Whiteside T. L. (2012). Myeloid-derived suppressor cell measurements in fresh and cryopreserved blood samples. *Journal of Immunological Methods*.

[30] Zeng Q.-L., Yang B., Sun H.-Q. (2014). Myeloid-derived suppressor cells are associated with viral persistence and downregulation of TCR *ζ* chain expression on CD8^+^ T cells in chronic hepatitis C patients. *Molecules and Cells*.

